# BeamNet: Unsupervised Beamforming for ISAC Systems Under Imperfect CSI

**DOI:** 10.3390/e28020175

**Published:** 2026-02-03

**Authors:** Helitha Nimnaka, Samiru Gayan, Ruhui Zhang, Hazer Inaltekin, H. Vincent Poor

**Affiliations:** 1 Department of Electronic and Telecommunication Engineering, University of Moratuwa, Katubedda 10400, Sri Lanka; nimnakakwh.20@uom.lk (H.N.); samirug@uom.lk (S.G.); 2 Institute of Advanced Study in Mathematics, Harbin Institute of Technology, Harbin 150001, China; ruhui_zhang@hit.edu.cn; 3 School of Engineering, Macquarie University, Sydney, NSW 2109, Australia; 4 Department of Electrical and Computer Engineering, Princeton University, Princeton, NJ 08544, USA; poor@princeton.edu

**Keywords:** integrated sensing and communication (ISAC), beamforming, unsupervised deep learning, imperfect CSI, Nakagami-*m* fading

## Abstract

Integrated sensing and communication (ISAC) is expected to be a key enabler for future wireless networks, improving spectral and hardware efficiency by jointly performing radar sensing and wireless communication within a unified framework. This paper proposes *BeamNet*, an unsupervised deep learning framework for transmit beamforming in dual-function radar-communication systems operating over general fading with imperfect channel state information (CSI). *BeamNet* maps noisy estimates of the communication and sensing channels to a transmit beamforming vector and is trained end-to-end by maximizing a weighted sum of the communication rate (CR) and sensing rate (SR), thereby learning the CR–SR Pareto frontier without beamforming labels or embedded optimization solvers. Using Rayleigh fading with perfect CSI, we first show that *BeamNet* reproduces the analytical Pareto-optimal beamforming solutions. We then use *BeamNet* to characterize, for Nakagami-*m* and Rician fading, the CR–SR trade-off across a range of fading parameters, and to assess robustness under distribution mismatch between training and test channels. Finally, under imperfect CSI, we demonstrate that *BeamNet* yields CR–SR trade-offs that are consistently sandwiched between the perfect-CSI and mismatched analytical baselines, outperforming the closed-form beamformer applied to imperfect CSI and recovering part of the performance loss caused by channel estimation errors. These results indicate that unsupervised learning offers a flexible and robust approach to ISAC beamforming in fading environments with imperfect channel knowledge.

## 1. Introduction

Next-generation wireless networks are increasingly expected not only to communicate but also to sense their surroundings in real time. Examples include vehicles detecting obstacles while exchanging safety messages and base stations tracking users and objects while serving data traffic. Integrated sensing and communication (ISAC) has emerged as a key paradigm to meet this demand by enabling joint radar sensing and wireless communication over shared spectral and hardware resources [1]. By tightly integrating these functions, ISAC can improve spectral efficiency, reduce latency, and simplify hardware, making it attractive for applications such as autonomous driving, smart transportation, urban infrastructure monitoring, and industrial automation. Unlike conventional frequency-division schemes that allocate separate resources to communication and sensing, ISAC leverages advanced signal processing and joint transceiver design to unify these tasks within a common framework [2].

Beamforming lies at the heart of ISAC, directly shaping both the communication rate (CR) and sensing rate (SR) (In this work, we adopt the SR, as it not only provides an information-theoretic metric directly comparable to the CR for unified CR–SR trade-off analysis, but also captures the fundamental information acquisition capability of the sensing process in ISAC systems). Striking the right balance between these inherently competing objectives is a fundamental yet challenging task, particularly as channel models and hardware constraints become more sophisticated. Existing research has largely focused on deriving analytical beamforming solutions under specific fading distributions. Notably, closed-form Pareto-optimal solutions are currently available only under perfect channel state (CSI) [3], while the practically relevant case of imperfect CSI remains analytically intractable. This gap is further compounded when considering more general fading environments, where exact distributional assumptions may be unknown or mismatched.

These limitations motivate a data-driven alternative for ISAC beamforming in non-ideal conditions. Rather than relying on closed-form designs that assume perfect CSI and specific fading laws, a deep learning (DL)-based approach can learn the mapping from observed (imperfect) channel realizations to beamforming vectors directly from data and then apply it in real time with low online complexity. Unsupervised learning is particularly appealing in this context: by optimizing task-oriented objectives (here, CR and SR) directly, it bypasses the need for explicit beamforming labels or embedded optimization solvers and can naturally accommodate diverse fading environments [4].

In this paper, we introduce *BeamNet*, an unsupervised learning framework for transmit beamforming in dual-function radar-communication ISAC systems operating over general fading channels with imperfect CSI. *BeamNet* takes noisy estimates of the communication and sensing channels as input and outputs a beamforming vector that balances CR and SR. The model is trained end-to-end using an unsupervised loss that maximizes a weighted sum of CR and SR, thereby tracing the CR-SR Pareto frontier without requiring hand-crafted labels or closed-form beamforming rules.

The key contributions of this work are as follows:

- We propose an unsupervised learning-based beamforming framework for ISAC systems operating under imperfect CSI, formulating beamforming as a multi-objective optimization problem in terms of CR and SR.
- The proposed approach is inherently distribution-agnostic at the loss level and can be trained on data from general fading channels (*BeamNet* is not tied to any specific distribution and only requires sample channels.) (e.g., Nakagami-*m*, Rician), enabling robust performance even under distribution mismatch between training and test channel statistics.
- Using a single trained model and by sweeping the loss weights, *BeamNet* efficiently characterizes the Pareto-optimal CR-SR trade-off across fading environments, providing a flexible tool to study ISAC performance frontiers beyond analytically tractable regimes.

Using numerical simulations, we first benchmark *BeamNet* against the analytical Pareto-optimal beamforming solution in [3] under Rayleigh fading with perfect CSI, and show that the learned CR-SR boundary closely reproduces the theoretical one. We then employ *BeamNet* to characterize the CR-SR Pareto trade-off under Nakagami-*m* fading and to assess robustness under distribution mismatch between training and testing channel statistics. Finally, under imperfect CSI, we demonstrate that *BeamNet* yields CR-SR trade-offs that lie between the analytical perfect-CSI upper bound and its imperfect-CSI counterpart, while consistently outperforming the closed-form beamformer obtained by directly applying the perfect-CSI solution to imperfect CSI estimates. These results indicate that unsupervised learning offers a practical and robust approach to ISAC beamforming in realistic fading environments with imperfect channel knowledge.

## 2. Related Work

Deep learning (DL) has emerged as a powerful tool for tackling key ISAC challenges, including mutual interference, high optimization complexity, and the need for real-time adaptability [5,6,7]. In [5], the authors present DL-based optimization techniques to maximize the weighted sum of sensing and communication rates in uplink 6G networks, achieving substantial real-time gains over conventional optimization methods. In [6], an end-to-end autoencoder-based framework jointly optimizes sensing accuracy, angle estimation, and communication quality, demonstrating resilience to hardware impairments, while [7] extends this to a semi-supervised design that reduces the reliance on labeled data. Predictive beamforming has also been explored; for example, ref. [8] employs a convolutional LSTM-based strategy for vehicular ISAC, predicting beamformers from historical CSI to reduce signaling overhead with performance close to ideal CSI. Beyond conventional communication-centric settings, Li et al. [9] propose an ISAC-based UWB system for fall detection (FallDR), showcasing ISAC’s potential for practical sensing applications.

Beyond supervised and end-to-end autoencoder-based designs, several recent works have explored *unsupervised* learning for ISAC beamforming. Ye et al. propose a lightweight IBF-Net architecture for RIS-aided ISAC, where image-shaped channel samples are processed, and an unsupervised loss jointly accounts for communication and sensing performance without requiring labeled beamformers [10]. Elrashidy et al. develop an unsupervised teacher-student framework for distributed beamforming in cell-free ISAC, dynamically balancing sensing signal-to-noise ratio (SNR) and communication signal-to-noise-plus-interference ratio (SINR) while reducing fronthaul overhead and computational complexity [11]. Temiz and Masouros present an unsupervised deep learning-based ISAC precoder that optimizes joint sensing and communication performance without requiring labeled precoders [4]. Together, these studies demonstrate the potential of label-free optimization for joint sensing and communication. However, they are tailored to specific architectures and do not explicitly characterize the CR-SR Pareto frontier under imperfect CSI.

Learning-based ISAC beamforming has also been investigated for large-scale and cell-free architectures. Demirhan and Alkhateeb propose a graph neural network (GNN)-based framework for cell-free massive MIMO ISAC, showing that heterogeneous GNNs can scale with the number of access points while approaching optimization-based performance without retraining for each topology [12]. This highlights the scalability of ML-driven beamforming, though their approach primarily relies on (near-)supervised targets derived from conventional optimization and pays limited attention to robustness under channel estimation errors.

Robust ISAC transceiver and beamforming designs under imperfect CSI have also attracted significant interest, predominantly using optimization-based (non-learning) methods. Zhang et al. investigate robust transceiver design for covert ISAC with imperfect CSI using bounded and probabilistic error models, and derive worst-case and outage-constrained solutions via S-procedure and Bernstein-type inequalities within an alternating optimization framework [13]. Chen et al. study robust beamforming for secure near-field ISAC systems, maximizing worst-case sensing beam pattern gain while satisfying SINR constraints for legitimate users and limiting information leakage under CSI uncertainty [14]. These methods provide strong robustness guarantees but depend on accurate error bounds and require repeatedly solving complex optimization problems, which can hinder real-time deployment, especially under distribution mismatch.

On the fundamental limits side, Guo et al. characterize the Pareto boundary of the CRB-rate tradeoff in ISAC with arbitrary input distributions using an information-theoretic (non-learning) framework [15], offering a complementary perspective and emphasizing the importance of explicitly tracing performance frontiers (CRB-rate or CR-SR) rather than optimizing a single objective. In a related direction, Zhang et al. introduce the Semi-Integrated-Sensing-and-Communication (Semi-ISAC) framework, evolving from OMA to NOMA and analyzing ergodic communication rate and radar estimation information rate over Nakagami-*m* fading channels [16]. This further motivates studying ISAC strategies over generalized fading environments beyond the standard Rayleigh model.

Compared with the above literature, the proposed *BeamNet* differs in three key aspects. First, unlike supervised, semi-supervised, or optimization-aided learning approaches that rely on target beamformers or near-optimal solutions, *BeamNet* is trained with an *unsupervised* objective directly defined in terms of the communication rate and sensing rate. This eliminates the need for explicit beamforming labels or embedded optimization solvers. Second, in contrast to robust but purely optimization-based ISAC designs and fundamental-limit analyses that require explicit error models or tractable fading assumptions, *BeamNet* is inherently *distribution-agnostic*: the same framework operates over general fading channels, including Nakagami-*m*, without relying on closed-form beamforming solutions or restrictive channel statistics. Third, while existing unsupervised ISAC beamforming methods demonstrate the promise of label-free optimization in specific architectures, they do not explicitly characterize the CR-SR Pareto frontier under imperfect CSI. In contrast, *BeamNet* incorporates *imperfect CSI* directly into the learning process and is used as a tool to trace the CR-SR Pareto boundary under channel estimation errors, thereby bridging analytically derived ideal-CSI designs and practical, data-driven beamforming in realistic fading and estimation conditions.

Beyond ISAC, related multi-functional designs arise in wireless communications frequently, involving coupled trade-offs such as rate-covertness in covert communications and rate-energy in simultaneous wireless information and power transfer (SWIPT) [17,18]. For example, Kang et al. in [17] study covert transmission limits in random-access networks and characterize achievable covert rates under covertness constraints in the presence of randomly activated overt users. In SWIPT-enabled MC-NOMA, Tang et al. [18] formulate joint power splitting and allocation to maximize data rate subject to energy-harvesting requirements, and additionally develop a learning-based approximation approach to reduce online complexity. While these works address different system models and objectives than ISAC beamforming, they illustrate the broader applicability of learning/optimization frameworks for rate-centric trade-offs. Extending *BeamNet* to these scenarios would require redefining the task-specific utility functions and constraints and is left for future work.

## 3. System Setup

**Figure 1:**
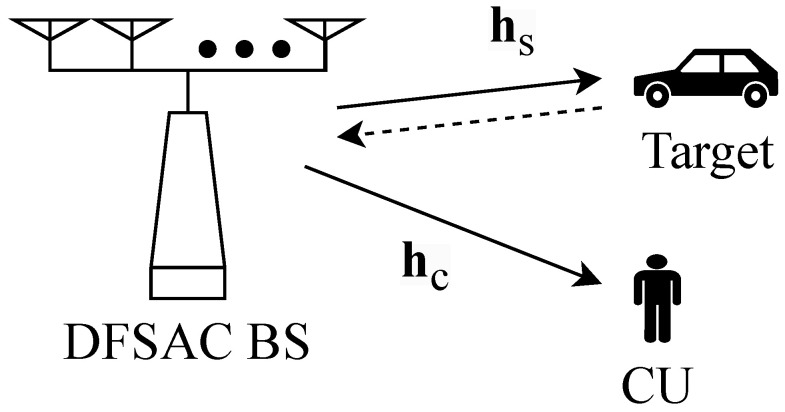
An ISAC system with a single antenna communication user and a single target.

We consider an ISAC system as shown in Figure 1, where a dual-function radar communication (DFRC) base station (BS), equipped with *M* transmit, and *N* receive antennas, simultaneously serves a single-antenna communication user (CU) and detects a single target. This setup aligns with the model presented in [3], which captures the use of shared hardware resources for joint communication and sensing. In this work, we adopt and build upon this setup to develop our learning-based framework.

Let $X=\left[x_{1}\ldots x_{L}\right]\in C^{M\times L}$ represents the DFRC signal matrix, where *L* denotes the length of the ISAC frame (consisting of communication symbols and the sensing pulses). From a communication point of view, $x_{l}\in C^{M}$ for l∈L={1,…,L} signifies the vector of the *l*-th data symbol. For sensing, $x_{l}$ corresponds to the sensing snapshot transmitted in the *l*-th time slot.

We consider the ISAC signal matrices in the form $X=\sqrt{p}ws^{H}$, where *p* denotes the power budget, $w\in C^{M}$ is the normalized beamforming vector and $s\in C^{L}$ represents the unit power data stream intended for the CU with $L^{-1}{∥s∥}^{2}=1$.

### 3.1. Communication Model

The signal received at the CU can be written as follows:

(1) $$y_{c}^{H}=\sqrt{p}h_{c}^{H}ws^{H}+n_{c}^{H}\in C^{1\times L},$$

where $n_{c}\in C^{L}$ is the additive noise with $n_{c}∼\mathrm{CN}\left(0,I\right)$, and $h_{c}\in C^{M}$ represents the state of the communication channel, which determines the quality of the communication link as shown in Figure 1. The CR, given $h_{c}$, satisfies

(2) $$\begin{array}{c} R_{c}=\log_{2}\left(1+p\right|w^{H}h_{c}{|}^{2}). \end{array}$$

We model the communication channel estimate to be imperfect according to

(3) $$\begin{array}{cc} h_{c} & =h_{c,\;\mathrm{perfect}}+e_{c}, \end{array}$$

where $h_{c,\;\mathrm{perfect}}$ denotes the true (perfect) communication channel and $e_{c}∼\mathrm{CN}\left(0,\sigma_{h}^{2}I\right)$ represents the channel estimation error.

### 3.2. Sensing Model

The DFRC BS observes the reflected echo signal at its receiver to sense the target. In particular, in response to X, the BS receives the echo

(4) $$\begin{array}{c} Y_{s}=\sqrt{p}Gws^{H}+N_{s}\in C^{N\times L}, \end{array}$$

where $N_{s}\in C^{N\times L}$ is the noise matrix with each entry having zero mean and unit variance, and $G\in C^{N\times M}$ represents the target response matrix. The target response matrix is modeled according to

(5) $$\begin{array}{c} G=\beta a\left(\theta \right)b^{H}\left(\theta \right), \end{array}$$

where β represents the radar cross section (RCS) of the target, which follows a complex Gaussian distribution CN(0,Γ) with Γ representing the average reflected signal power. The vectors $a\left(\theta \right)\in C^{N}$ and $b\left(\theta \right)\in C^{M}$ correspond to the receive and transmit array steering vectors, respectively, as a function of the target angle θ [3,19].

We model the antenna aperture at the BS using a uniform linear array with half-wavelength spacing, which yields

(6) $$a\left(\theta \right)={\left[e^{j\pi (n-1)\sin\theta }\right]}_{n=1}^{N},\;b\left(\theta \right)={\left[e^{j\pi (m-1)\sin\theta }\right]}_{m=1}^{M}.$$

We adopt the Swerling-I model [20] to compute the sensing rate, which assumes that a target’s RCS remains constant within each pulse transmission but varies stochastically from pulse to pulse (according to the assumed distribution for β). Under this model, for a given w and using matched-filter receiver beamforming matched to a(θ), the sensing rate $R_{s}$ can be approximated by

(7) $$\begin{array}{c} R_{s}=L^{-1}\log_{2}\left(1+pNL\Gamma |w^{H}h_{s}{|}^{2}\right), \end{array}$$

where $pNL\Gamma |w^{H}h_{s}{|}^{2}$ is the average *sensing* SNR and $h_{s}≜b\left(\theta \right)$ is the sensing channel.

**Remark (SR vs. radar KPIs):** While the sensing rate $R_{s}$ in Equation (7) is an information-theoretic metric (rather than a detector-specific KPI), it is closely aligned with classical sensing performance measures under the adopted Swerling-I and matched-filter processing model. In particular, Equation (7) is a monotone function of the average sensing SNR, ${\mathrm{SNR}}_{s}=pNL\Gamma {\left|w^{H}h_{s}\right|}^{2}$, so that maximizing $R_{s}$ is equivalent to maximizing ${\mathrm{SNR}}_{s}$ for fixed (p,N,L,Γ). Since standard radar detection probability at a fixed false-alarm rate (Neyman–Pearson testing) and typical estimation accuracy trends (e.g., CRB/RMSE behavior) improve monotonically with sensing SNR, improvements in $R_{s}$ translate into improved detection/estimation capability once a specific detector/estimator and operating point are chosen. Moreover, the mapping is explicit: ${\mathrm{SNR}}_{s}=2^{LR_{s}}-1$, enabling direct conversion from the CR-SR frontier to SNR-based radar KPIs when desired.

The target’s angle θ is estimated by high-resolution direction-of-arrival algorithms, such as MUSIC or Capon, which process the received echo to extract phase differences across antenna elements in the steering vector a(θ) [21]. We assume the target angle estimation is imperfect, modeled according to

(8) $$\begin{array}{cc} \theta & =\theta_{\mathrm{perfect}}+e_{s}, \end{array}$$

where $\theta_{\mathrm{perfect}}$ denotes the true target angle and $e_{s}∼N\left(0,\sigma_{s}^{2}\right)$ represents the estimation error in the target angle.

Given the above ISAC framework, we aim to analyze the joint sensing and communication performance of the BS.

Note that both $R_{c}$ and $R_{s}$ are influenced by the beamforming vector w. Finding an optimal w that maximizes both $R_{c}$ and $R_{s}$ is a multi-objective optimization problem. There are commonly three design paradigms to investigate this problem: (i) communication-centric (C-C), (ii) sensing-centric (S-C), and (iii) Pareto-optimal design.

Despite the elegant structure of the above ISAC model, obtaining closed-form beamforming solutions is generally tractable only under idealized assumptions. For instance, when assuming perfect CSI, ideal synchronization, and absence of hardware impairments, the Pareto-optimal beamforming strategies and the resulting CR–SR trade-offs can be analytically derived, as demonstrated in [3,22]. However, when departing from this ideal regime, such as in the presence of imperfect channel estimation or hardware impairments, the resulting optimization problem becomes analytically intractable. In particular, robust beamforming under imperfect CSI often involves computing expectations over uncertain or mismatched error statistics, and simultaneously optimizing the conflicting CR and SR objectives under these uncertainties further complicates the overall design. As a result, direct extensions of perfect-CSI analytical solutions often yield suboptimal or overly conservative beamformers when applied in realistic conditions.

These limitations motivate a data-driven alternative for beamforming under non-ideal conditions. Rather than relying on closed-form analytical solutions that assume perfect CSI, a DL–based approach can learn the mapping from observed imperfect channel realizations to beamforming vectors directly from data. This strategy offers three major advantages. First, it can be trained to remain robust against CSI imperfections by exposing the model to estimation errors during training. Once trained, it enables real-time beamformer computation with low online complexity. Last, it is well-suited for fast-varying ISAC scenarios in diverse fading environments. In this paper, we introduce *BeamNet*, an unsupervised DL framework that directly optimizes a CR–SR objective, learns optimal beamforming under imperfect CSI, and generalizes across a broad range of fading conditions beyond the capabilities of existing analytical designs.

## 4. DL-Based Beamforming Framework

### 4.1. Deep Learning Model Architecture

**Figure 2:**
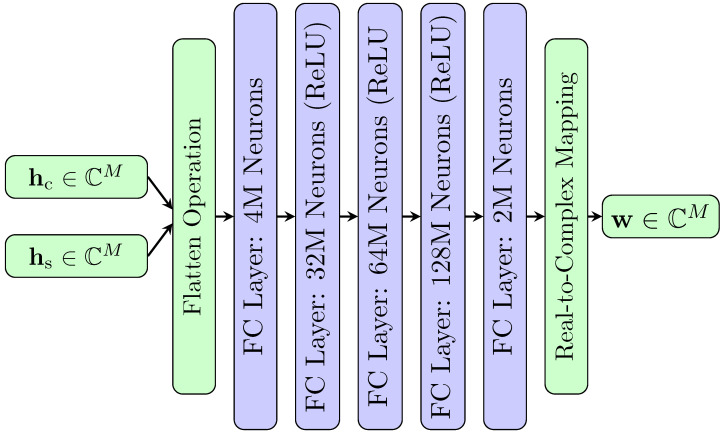
The architecture of the *BeamNet* deep learning model.

First, we provide an overview of our *BeamNet* architecture, as illustrated in Figure 2, that jointly maximizes CR and SR. In our proposed architecture, the input data and the communication channel $h_{c}\in C^{M}$ and sensing channel $h_{s}\in C^{M}$ go through a flattening operation to divide into real and imaginary parts to process complex-valued inputs. The model predicts 2M outputs, which are then mapped to the real and imaginary components of the beamforming vector $w\in C^{M}$.

The architecture employs fully connected layers with Rectified Linear Unit (ReLU) activations to map the concatenated input features to the desired beamforming vector. The model processes the input through a hierarchy of layers, progressively learning the non-linear relationship between the channels and the beamforming weights.

### 4.2. Deep Learning Model Training

We use the following loss function to train the *BeamNet*

(9) $$\begin{array}{cc} L & =-\left(\left(1-\alpha \right)\cdot R_{c}+\alpha \cdot R_{s}\right), \end{array}$$

where α∈[0,1] controls the CR–SR trade-off: α=0 yields a communication-centric model (maximizing $R_{c}$), α=1 yields a sensing-centric model (maximizing $R_{s}$), and intermediate values balance the two objectives.

BeamNet takes the imperfect CSI estimates $\left(h_{c},h_{s}\right)$ as input. During training on synthetic data, the loss in Equation (9) is evaluated using the underlying true channels $\left(h_{c,\mathrm{perfect}},h_{s,\mathrm{perfect}}\right)$ to provide stable gradients and to explicitly learn a mapping from noisy CSI to a beamformer that maximizes the true CR and SR.

To facilitate reproducibility, Table 1 summarizes the training and implementation settings. We generate $10^{5}$ independent channel realizations following the fading model under consideration (Nakagami-*m* or Rician), and draw $\theta_{\mathrm{perfect}}∼U\left[0,\pi \right]$ to construct the sensing channel. The dataset is split into 85% for training and 15% for testing.

For each scalarization weight α, the network parameters are trained for 500 epochs using Adam with learning rate $10^{-3}$ and full-batch updates over the training set. We use PyTorch’s default Kaiming-uniform initialization for linear layers and do not apply explicit $L_{1}$/$L_{2}$ regularization or weight decay. The CR-SR Pareto boundary is obtained by sweeping α over 101 uniformly spaced values in [0,1] (step size 0.01). Unless otherwise stated, all real-valued tensors and parameters use 32-bit floating point precision (FP32), while complex-valued channel variables are stored as complex64. The resulting model requires approximately $1.70\times 10^{5}$ FLOPs per inference (per channel realization) and achieves an average inference latency of 0.8694 ms ±0.0344 ms on an NVIDIA GeForce RTX 2080 Ti (CUDA 12.7).

In Section 5.4, we evaluate robustness by varying the channel estimation error variance $\sigma_{h}^{2}$ and the target angle estimation error variance $\sigma_{s}^{2}$ over the range [0.001,0.1] and reporting the corresponding CR-SR trade-offs.

**Remark-Synthetic dataset generation and reproducibility:** All training/test samples are generated using Monte Carlo simulation from the statistical models specified in Section 3 and Section 5. For each realization, we draw a true communication channel $h_{c,\mathrm{perfect}}$ from the stated fading distribution (e.g., Nakagami-*m*: $h_{c,i}=r_{i}e^{j\phi_{i}}$ with $r_{i}∼\mathrm{Nakagami}\left(m,\Omega \right)$ and $\phi_{i}∼U\left[0,2\pi \right)$), draw $\theta_{\mathrm{perfect}}∼U\left[0,\pi \right]$ to construct the sensing channel, and then generate imperfect CSI using the estimation-error models with variances $\sigma_{h}^{2}$ and $\sigma_{s}^{2}$. Thus, given the reported parameter settings (Table 1 and Section 5), the full dataset and all results are reproducible without relying on any external dataset.

## 5. Experimental Results

In this section, we present numerical results evaluating the performance of the proposed ISAC *BeamNet* beamformer. The proposed framework is inherently independent of the underlying channel fading distribution. To validate its accuracy, we first consider a Rayleigh fading channel and benchmark our results against the analytical solution in [3] under perfect channel conditions. We then extend the evaluation to Nakagami-*m* fading under perfect channel conditions.

The Nakagami-*m* fading channel was considered in this paper because it offers a versatile and general framework for modeling a wide variety of wireless propagation conditions. Specifically, it reduces to Rayleigh fading for m=1, can approximate Rician fading for m>1, and captures more severe fading than Rayleigh when 0.5≤m<1 [23]. This flexibility allows it to represent diverse practical scenarios such as urban microcells, indoor channels, or obstructed environments, where different fading severities may occur. Owing to these properties, Nakagami-*m* is widely used in the literature as a fading channel model. In our work, its inclusion enables us to evaluate the robustness of the proposed method across a broad range of fading conditions, rather than restricting the analysis to a single channel model.

To that end, the channel fading vector $h_{c,\mathrm{perfect}}\in C^{M}$ is modeled as a multi-dimensional Nakagami-*m* random vector, where each entry ${\left[h_{c,\mathrm{perfect}}\right]}_{i}$ is independently drawn from a Nakagami-*m* distribution with shape parameter *m* and scale parameter Ω, expressed as ${\left[h_{c,\mathrm{perfect}}\right]}_{i}=r_{i}e^{j\phi_{i}},$ where $r_{i}∼\mathrm{Nakagami}\text{-}m\left(m,\Omega \right)$ and $\phi_{i}∼U\left[0,2\pi \right)$.

We present the Pareto-optimal CR-SR trade-off boundaries for both Rayleigh fading and general Nakagami-*m* fading scenarios. Unless otherwise stated, the main simulation parameters are: M=4, N=5, L=20, m∈{0.5,1,2,3}, and Ω=Γ=0.95.

After benchmarking our results with respect to the theoretical optimum, we present numerical evaluations under imperfect CSI conditions, considering Nakagami-*m* fading. To this end, we set $\sigma_{h}=\sigma_{s}=$ 0.05 and 0.1 and demonstrate that the proposed DL-based framework outperforms the analytical framework under imperfect channel conditions.

### 5.1. Benchmark BeamNet Under Rayleigh Fading (m=1) with Perfect CSI

In this section, we consider perfect channel conditions, where $h_{c}=h_{c,\;\mathrm{perfect}}∼\mathrm{CN}\left(0,I\right)$ and $\theta =\theta_{\mathrm{perfect}}∼U\left[0,\pi \right]$.

#### 5.1.1. Communication-Centric Design

In this scenario, the optimum beamforming vector $w_{c}$ is given by [3],

(10) $$\arg\max_{w}R_{c}=\arg\max_{w}\left|w^{H}h_{c}\right|={∥h_{c}∥}^{-1}h_{c}≜w_{c}.$$

**Figure 3:**
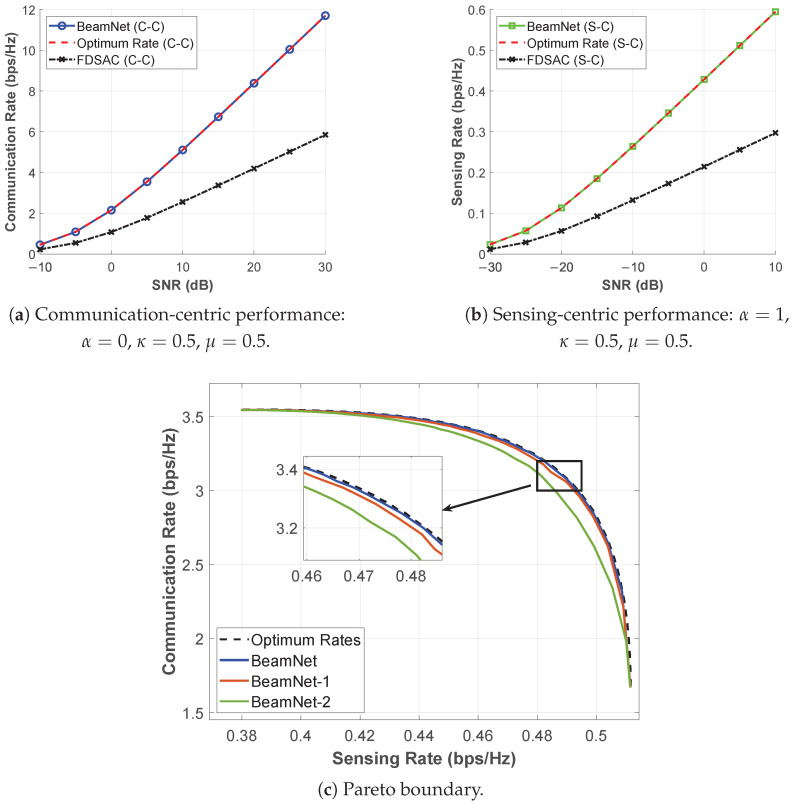
Benchmarking the performance of *BeamNet* under Rayleigh fading at SNR=5 dB. *BeamNet*-1 and *BeamNet*-2 are depth-ablation variants (reduced-capacity baselines).

Using Equation (10) as a benchmark giving the optimum beamforming vector for communication, we provide the performance of *BeamNet* in Figure 3a. The results show that *BeamNet* attains a communication rate almost identical to the analytical optimum reported in [3] under the C-C design.

#### 5.1.2. Sensing-Centric Design

In this scenario, the optimum beamforming vector $w_{s}$ is given by [3],

(11) $$\arg\max_{w}R_{s}=\arg\max_{w}\left|w^{H}h_{s}\right|={∥h_{s}∥}^{-1}h_{s}≜w_{s}.$$

Using Equation (11) as a benchmark giving the optimum beamforming vector for sensing, we provide the performance of *BeamNet* in Figure 3b. The results show that *BeamNet* attains a sensing rate almost identical to the analytical optimum reported in [3] under the S-C design.

#### 5.1.3. Pareto Optimal Design

In this scenario, the optimum beamforming vector w is obtained by solving

(12) $$\begin{array}{c} \max_{w,R}\;R,s.t.R_{s}\geq \alpha R,R_{c}\geq \left(1-\alpha \right)R,{∥w∥}^{2}=1, \end{array}$$

where α∈[0,1] is a rate-profile parameter and *R* is the optimization objective, which represents a trade-off between communication rate and sensing rate [3,22,24].

As presented in [3], the Pareto boundary of the rate region can be attained using the beamforming vector,

(13) $$\begin{array}{c} w_{\xi }=\frac{\left(1-\xi \right)h_{c}+\xi h_{s}e^{-j∠\alpha_{12}}}{∥\left(1-\xi \right)h_{c}+\xi h_{s}e^{-j∠\alpha_{12}}∥}, \end{array}$$

where $\alpha_{12}=p\sqrt{NL\Gamma h_{c}^{H}h_{s}}$ and the weighting factor ξ varies within the range [0,1].

To discover the Pareto boundary achieved by *BeamNet*, we increment the hyperparameter α∈[0,1] with a step size of 0.01. Figure 3c shows that the Pareto boundary obtained using the *BeamNet* closely follows the one obtained with Equation (13).

We further use frequency-division sensing-and-communication (FDSAC) as another benchmark to compare the performance achieved by *BeamNet*. The FDSAC scheme is adopted as a conventional baseline, serving to illustrate the performance gain achievable through integration in ISAC systems. For the FDSAC case, the CR and the SR are given by $R_{c}^{f}=\kappa \log_{2}\left(1+\frac{\mu }{\kappa }p{∥h_{c}∥}^{2}\right)$ and $R_{s}^{f}=\frac{1-\kappa }{L}\log\left(1+\frac{1-\mu }{1-\kappa }pNL\Gamma {∥h_{s}∥}^{2}\right)$, where μ and κ are the fractions of power and bandwidth dedicated to the communication task, respectively [3].

In summary, Figure 3 shows that *BeamNet* consistently achieves performance close to the analytical optimum under Rayleigh fading across all three considered cases, confirming its effectiveness.

#### 5.1.4. Impact of the Number of Layers (Ablation Study)

To isolate the impact of network depth, we conduct a depth ablation study in Figure 3c. The performance of *BeamNet* improves and approaches the analytical Pareto boundary in [3] as the number of hidden layers (and thus model capacity) increases. Specifically, Figure 3c compares the full *BeamNet* with two deliberately reduced-capacity variants: *BeamNet-1*, obtained by removing the 128M hidden layer, and *BeamNet-2*, obtained by removing both the 64M and 128M hidden layers. *These variants are included only as ablation baselines (not as recommended architectures). Hence, their lower performance is expected and serves to quantify the depth-performance trade-off.* The results show that increasing depth systematically improves the learned CR-SR boundary (*BeamNet*-2 → *BeamNet*-1 → *BeamNet*). Based on extensive experiments, we adopt the architecture in Figure 2, which delivers near-optimal performance with moderate computational complexity. Increasing width or depth beyond this point yields negligible gains at higher computational cost.

### 5.2. Performance Under Nakagami-m Fading with Perfect CSI

Next, we examine the impact of Nakagami-*m* fading on *BeamNet*’s performance by analyzing the $R_{c}$–$R_{s}$ trade-off under different fading conditions. Specifically, we evaluate four shape parameters, m∈0.5,1,2,3, while fixing the spread parameter at Ω=0.95.

**Figure 4:**
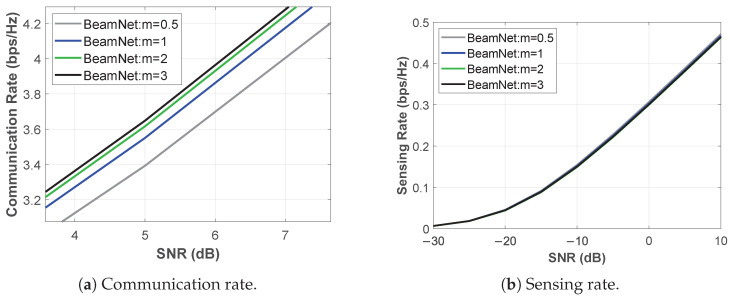
Performance under C-C design over Nakagami-*m* fading (α=0): (**a**) CR, (**b**) achieved SR.

**Figure 5:**
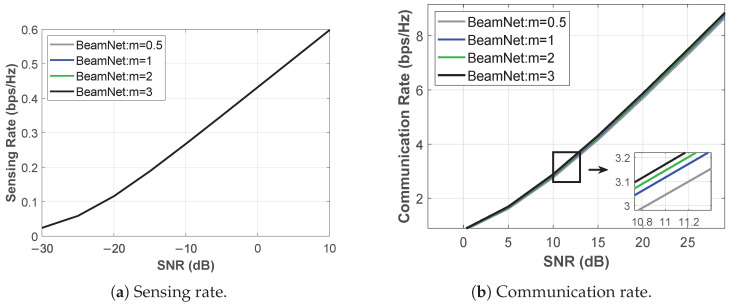
Performance under S-C design over Nakagami-*m* fading (α=1): (**a**) SR, (**b**) achieved CR.

**Remark-Why are both CR and SR reported in Figure 4 and Figure 5 below:** Although the C-C and S-C designs optimize a single objective (CR-only or SR-only), the resulting transmit beamformer w simultaneously shapes both links, and therefore induces a well-defined value for the other metric as well. For completeness, Figure 4 and Figure 5 report *both* the optimized rate and the corresponding non-optimized rate to quantify the dual-task performance at these two extreme operating points. These extreme points also serve as endpoint references that help interpret the Pareto-optimal CR-SR trade-offs reported in Section 5.2.3.

#### 5.2.1. Communication-Centric Design

Figure 4 illustrates the communication and sensing rates when the model is trained for the C–C design under Nakagami-*m* fading with m∈{0.5,1,2,3}. In this configuration, *BeamNet* optimizes the beamforming vector solely to maximize the CR, without explicitly accounting for the SR. For instance, at m=2 and SNR=5 dB, the CR reaches 3.616 bps/Hz, while the SR is 0.3814 bps/Hz. As expected, the CR improves with increasing *m*; however, the incremental improvement becomes less pronounced as the fading condition transitions from severe to nearly non-fading.

#### 5.2.2. Sensing-Centric Design

Figure 5 shows the communication and sensing rates when the model is trained for the S–C design under Nakagami-*m* fading with m∈{0.5,1,2,3}. In this setting, *BeamNet* optimizes the beamforming vector exclusively to maximize the SR, without explicitly considering the CR. For example, at m=2 and SNR=5 dB, the SR reaches 0.5153 bps/Hz, while the CR is 1.6799 bps/Hz. Compared to the C–C design results in Figure 4 for the same *m* and SNR, the SR is higher; however, the CR is lower.

#### 5.2.3. Pareto Optimal Design

**Figure 6:**
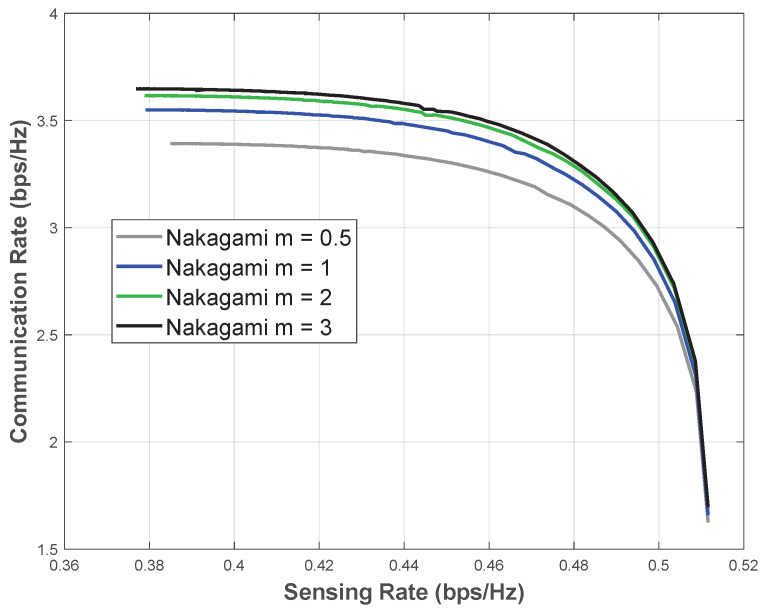
Pareto boundary under Nakagami-*m* fading with m=0.5,1,2,3 at SNR=5 dB.

Figure 6 presents the Pareto boundaries under Nakagami-*m* fading, where *BeamNet* jointly optimizes the beamforming vector to maximize both the CR and the SR. For example, at m=2 and SNR=5 dB, when operating in a communication-centric regime (small α), the CR reaches approximately 3.55 bps/Hz with an SR of about 0.38 bps/Hz, which is consistent with the C-C design in Figure 4. In contrast, for a sensing-centric setting (α=1), the SR attains its maximum value of $R_{s}\approx 0.512$ bps/Hz with a corresponding communication rate of $R_{c}\approx 1.66$ bps/Hz, in agreement with the S-C design in Figure 5. Moreover, for the same *m* and SNR, intermediate $\left(R_{s},R_{c}\right)$ operating points along the Pareto boundary can be achieved; for instance, $R_{s}\approx 0.44$ bps/Hz with $R_{c}\approx 3.48$ bps/Hz (e.g., α≈0.76) and $R_{s}\approx 0.48$ bps/Hz with $R_{c}\approx 3.20$ bps/Hz (e.g., α≈0.93).

**Figure 7:**
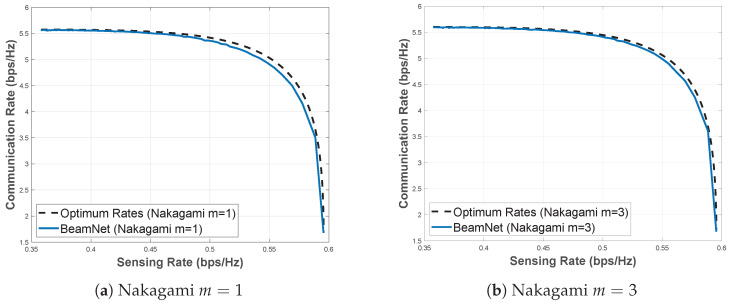
Pareto boundary under Nakagami-*m* fading with m=1,3 at SNR=5 dB, with 16 transmit antennas and 4 receive antennas.

*BeamNet* works with different transmit and receive antenna configurations. To demonstrate this, we evaluate a system with 16 transmit antennas and 4 receive antennas under Nakagami-*m* fading with m=1 and m=3, as shown in Figure 7. The results show that *BeamNet* achieves performance very close to the optimal rates. This confirms that *BeamNet* generalizes well across different antenna configurations.

### 5.3. Model Robustness Test and Generalization

**Figure 8:**
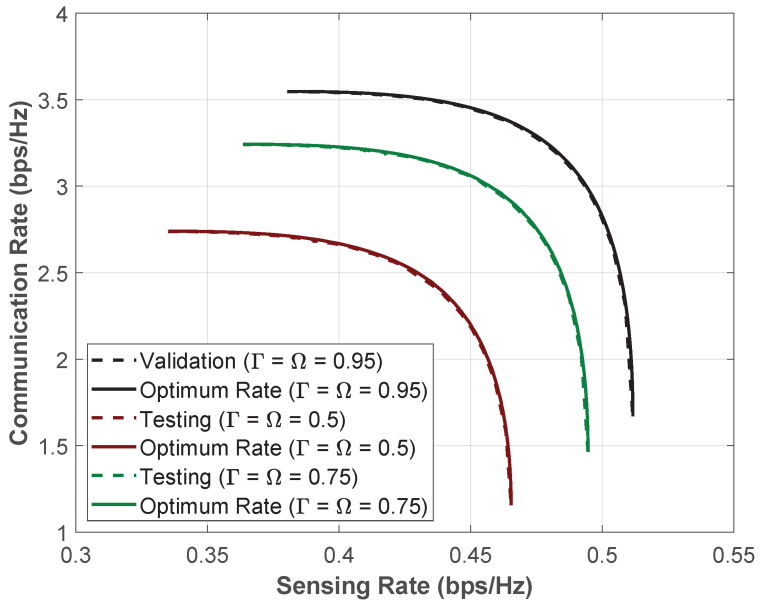
Model robustness across different channel variances.

Next, we evaluate the *BeamNet* robustness when the training data distribution does not exactly follow the test data distribution. Recall that we train the model using 85,000 data points, and we use 15,000 data points for model testing, both sampled from the same distribution with parameters Ω=Γ=0.95. To evaluate the model under varying channel conditions, we conduct tests using 15,000 data points per set, but with channel parameters Ω=Γ∈{0.5,0.75}. In Figure 8, we illustrate that *BeamNet* performs very close to the trade-off boundary even when the test data distribution differs from the distribution generating training and testing data.

This result demonstrates the robustness of *BeamNet* to variations in channel statistics between training and testing, and its ability to generalize beyond the training and testing parameters.

### 5.4. Performance Under Nakagami-m Fading with Imperfect CSI

Next, we analyze the effect of *imperfect channel estimation* on the CR-SR trade-off. It is important to note that the CR-SR Pareto boundary under imperfect CSI is not available in closed form. Hence, in this part of the paper, we assess the *BeamNet* performance using the analytical perfect-CSI Pareto boundary as an ideal upper performance bound and the closed-form beamformer computed from imperfect CSI estimates as a mismatched lower performance bound, as explained below.

During training, *BeamNet* receives noisy $\left(h_{c},h_{s}\right)$ as input, while the unsupervised loss in Equation (9) is evaluated using the *true*$\left(h_{c,\mathrm{perfect}},h_{s,\mathrm{perfect}}\right)$ to stabilize learning and encourage robustness against estimation errors. During testing, the model is fed with $\left(h_{c},h_{s}\right)$, and the results are compared against the analytical beamforming vector derived from Equation (13) under perfect CSI conditions.

We obtain an upper bound on the *BeamNet* performance by substituting the perfect CSI $\left(h_{c,\mathrm{perfect}},h_{s,\mathrm{perfect}}\right)$ into Equation (13) (referred to as the *Analytical with Perfect CSI* bound), and a lower bound on the *BeamNet* performance by substituting the imperfect CSI $\left(h_{c},h_{s}\right)$ into Equation (13) (referred to as the *Analytical with Imperfect CSI* bound).

The reported performance results use single-precision floating point for all real-valued tensors and network parameters, while complex-valued channel variables are stored as complex64 (FP32 real and imaginary parts), as noted above. This is a standard and numerically stable setting for wireless channel modeling. Unless otherwise specified, we set SNR=5 dB, M=4, N=5, L=20, and Ω=Γ=0.95.

**Remark:** Although we present the main numerical results for a representative baseline configuration to enable direct comparison with analytical references, we also verified *BeamNet* under larger antenna arrays (e.g., M=16,N=4) and under more severe CSI errors beyond the nominal range. The same qualitative CR-SR trends persist, and *BeamNet* exhibits graceful degradation until the CSI becomes highly unreliable. These results are omitted for brevity.

**Figure 9:**
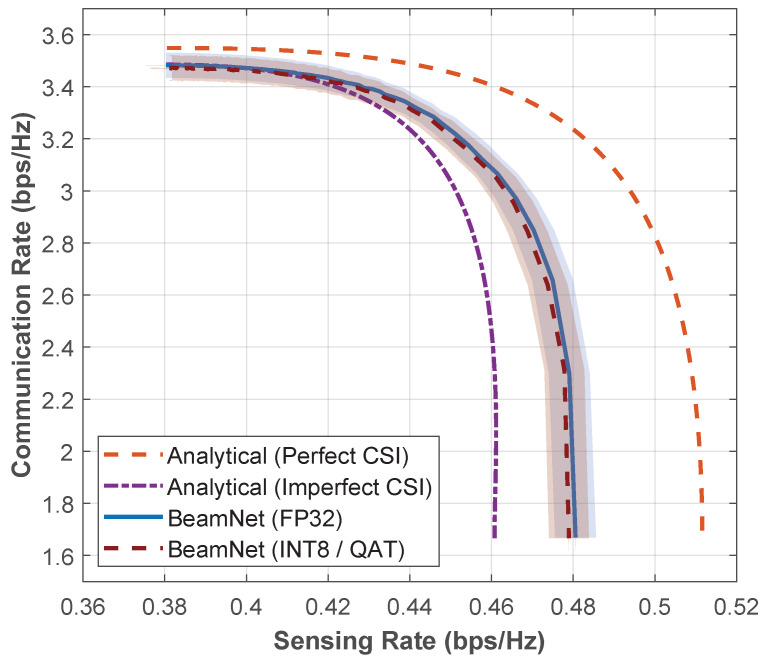
CR-SR Pareto boundary for Nakagami-*m* fading with m=1 (i.e., Rayleigh fading) at SNR=5 dB under imperfect CSI with $\sigma_{h}^{2}=\sigma_{s}^{2}=0.05$. Shaded region indicates the 95% confidence interval.

Figure 9 shows the CR-SR Pareto boundary under Rayleigh fading with $\sigma_{h}^{2}=\sigma_{s}^{2}=0.05$, where the *BeamNet* Pareto boundary lies between the *Analytical with Perfect CSI* upper-bound and the *Analytical with Imperfect CSI* lower-bound baselines, while remaining visibly closer to the perfect-CSI curve across most operating points. The shaded region represents the 95% confidence interval. The figure demonstrates that, even when operating solely with imperfect CSI inputs, *BeamNet* learns beamforming strategies that are significantly more robust than directly applying the analytical solution under imperfect CSI conditions. For moderate sensing rates around $R_{s}\approx 0.44$ bps/Hz, *BeamNet* achieves a communication rate that is approximately 0.3% higher than the *Analytical with Imperfect CSI* lower-bound, while remaining within about 3.5–4% of the *Analytical with Perfect CSI* upper-bound. This confirms that, even when only imperfect CSI is available at test time, the learned beamformer closely tracks the ideal Pareto boundary and avoids the degradation caused by directly using imperfect CSI in the closed-form solution provided in Equation (13).

From a deployment perspective, we also evaluate a hardware-constrained, low-precision implementation of *BeamNet* suitable for resource-limited processors and edge accelerators. To obtain a low-complexity model with a reduced memory footprint and efficient integer arithmetic support, we apply Quantization-Aware Training (QAT) and export an INT8 version of the network. Specifically, activations are quantized using per-tensor affine quantization (uint8, 0–255), and weights are quantized using per-channel symmetric quantization (int8, −128 to 127). This reduces parameter storage by approximately 75% compared to *BeamNet* FP32 implementation while preserving the same feed-forward inference structure. As shown in Figure 9, the INT8/QAT Pareto boundary closely tracks the full-precision boundary, indicating that the learned beamformer is robust to 8-bit quantization and remains effective under practical hardware constraints. For reference, the model requires approximately $1.70\times 10^{5}$ FLOPs per inference (per channel realization) in both cases.

**Figure 10:**
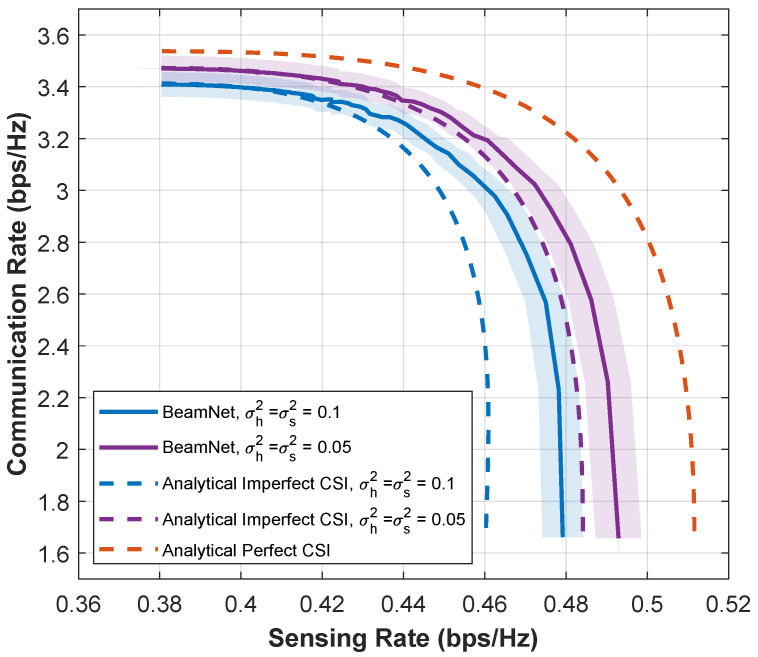
CR-SR Pareto boundaries for m=1 at SNR=5 dB under different estimation error variances $\sigma_{h}^{2}=\sigma_{s}^{2}\in \left\{0.05,0.10\right\}$. Shaded regions indicate 95% confidence intervals.

Figure 10 further illustrates the impact of estimation error variances on the CR-SR trade-off, along with 95% conficence intervals (shaded areas). As the error variances $\sigma_{h}^{2}$ and $\sigma_{s}^{2}$ increase from 0.05 to 0.1, the *Analytical with Imperfect CSI* curve deviates significantly from the *Analytical with Perfect CSI* curve in both CR and SR. In contrast, the *BeamNet* curve exhibits a more graceful degradation and remains consistently sandwiched between the two bounds, staying closer to the upper bound across all considered operating points and error levels. For example, at $R_{s}\approx 0.44$ bps/Hz and $\sigma_{h}^{2}=\sigma_{s}^{2}=0.10$, *BeamNet* improves the communication rate by approximately 3.2% compared to the mismatched analytical solution, while the gap to the perfect-CSI bound is about 6–7%. These results indicate that the benefit of *BeamNet* becomes more pronounced as CSI imperfections increase, effectively narrowing a substantial fraction of the performance loss introduced by estimation errors.

**Figure 11:**
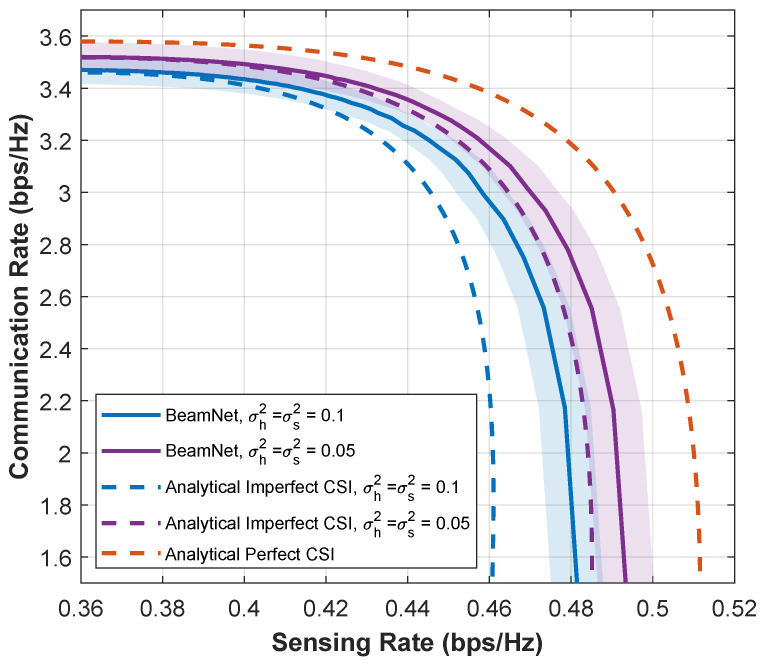
CR-SR Pareto boundaries for k=1 at SNR=5 dB under different estimation error variances $\sigma_{h}^{2}=\sigma_{s}^{2}\in \left\{0.05,0.10\right\}$. Shaded regions indicate 95% confidence intervals.

In Figure 11, we repeat the same uncertainty-quantification study under Rician-*k* fading (k=1), which introduces a Line-of-Sight (LoS) component in addition to scattered multipath. The shaded regions denote the 95% confidence intervals of the CR–SR operating points under imperfect CSI. Consistent with Figure 10, *BeamNet* continues to produce CR–SR trade-offs that remain between the analytical perfect-CSI upper bound and the mismatched analytical imperfect-CSI baseline, while the confidence bands remain relatively tight, indicating stable behavior across random channel and estimation-error realizations.

**Figure 12:**
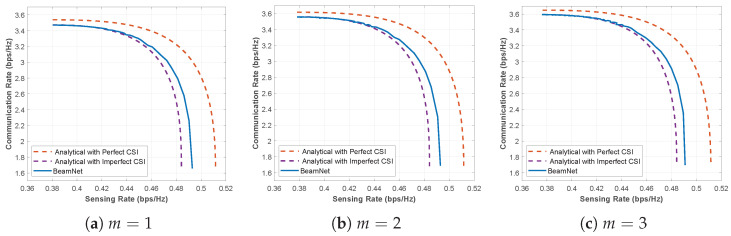
CR-SR Pareto boundary under imperfect CSI with $\sigma_{h}^{2}=\sigma_{s}^{2}=0.05$ for Nakagami-*m* fading with m∈{1,2,3} at SNR=5 dB.

We next assess the performance of *BeamNet* across different fading conditions by considering Nakagami-*m* fading with m∈{1,2,3} under imperfect CSI with $\sigma_{h}^{2}=\sigma_{s}^{2}=0.05$. The corresponding CR-SR curves are depicted in Figure 12. In all three cases, the *BeamNet* curve lies noticeably above the *Analytical with Imperfect CSI* baseline and remains close to the *Analytical with Perfect CSI* benchmark. Around a representative operating point with $R_{s}\approx 0.48$ bps/Hz, *BeamNet* achieves a communication rate of about $R_{c}\approx 2.79$, 2.87, and 2.91 bps/Hz for m=1, m=2, and m=3, respectively, compared to approximately $R_{c}\approx 1.75$–1.78 bps/Hz for the *Analytical with Imperfect CSI* design, corresponding to a relative $R_{c}$ gain on the order of 60–65% at the same sensing rate. This behavior demonstrates that the proposed unsupervised framework generalizes reliably across different channel conditions.

**Figure 13:**
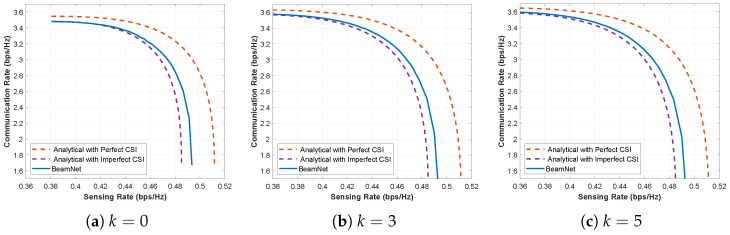
CR-SR Pareto boundary under imperfect CSI with $\sigma_{h}^{2}=\sigma_{s}^{2}=0.05$ for Rician-*k* fading with k∈{0,3,5} at SNR=5 dB.

To further support the distribution-agnostic nature of the proposed approach, we repeat the imperfect-CSI evaluation under Rician-*k* fading for different *k* values in Figure 13. This figure shows the resulting CR–SR Pareto boundaries for k∈{0,3,5} at SNR=5 dB with $\sigma_{h}^{2}=\sigma_{s}^{2}=0.05$. Consistent with the Nakagami-*m* case in Figure 12, *BeamNet* continues to yield trade-off curves that track the perfect-CSI benchmark substantially more closely than the mismatched analytical imperfect-CSI baseline, indicating that the same robustness trends persist in LoS-dominant propagation environments.

## 6. Conclusions and Future Work

In this paper, we introduced *BeamNet*, an unsupervised deep learning framework for transmit beamforming in ISAC systems operating over general fading channels with imperfect CSI. *BeamNet* maps noisy estimates of the communication and sensing channels to a beamforming vector and is trained end-to-end using a task-oriented loss that directly combines the communication rate (CR) and sensing rate (SR). By sweeping the loss weight, a single trained model yields the CR-SR Pareto frontier without requiring labeled beamformers or embedded optimization solvers.

Our numerical results show that, under Rayleigh fading with perfect CSI, *BeamNet* accurately reproduces the analytically derived Pareto boundary and matches the communication- and sensing-centric benchmarks, thereby validating the proposed learning framework against known optimal solutions. Extending to Nakagami-*m* fading, *BeamNet* efficiently traces CR-SR trade-off curves across a range of fading parameters and remains robust when the test-channel statistics differ from those seen during training, demonstrating its ability to generalize beyond a single channel law. Under imperfect CSI, *BeamNet* is trained and evaluated with noisy channel estimates and yields CR-SR trade-offs that are consistently sandwiched between the analytical perfect-CSI upper bound and the analytical beamformer applied to imperfect CSI, providing systematic gains over the latter across estimation error levels and fading conditions. Taken together, these results highlight *BeamNet* not only as a practical beamforming scheme under imperfect CSI, but also as a flexible numerical tool for exploring ISAC performance frontiers in regimes where analytical solutions are unavailable or intractable.

Future research directions include (i) extending *BeamNet* to *joint channel estimation and beamforming*, so that channel uncertainty is incorporated natively into the learning process; (ii) investigating beamforming under practical hardware impairments such as low-resolution analog-to-digital converters (ADCs) and digital-to-analog converters (DACs), nonlinear RF front-ends, and quantization constraints; and (iii) integrating synchronization-related imperfections (e.g., timing offsets and carrier-frequency offsets) into the end-to-end design. Collectively, these extensions will further tighten the achievable CR–SR Pareto frontier under realistic hardware and synchronization impairments and provide new design insights for deploying unsupervised-learning-based ISAC schemes in next-generation wireless systems.

## Figures and Tables

**Table 1:** *BeamNet* training protocol and implementation details.

Item	Setting
Dataset size	$10^{5}$ synthetic channel realizations
Train/Test split	85%/15% (85,000/15,000)
Batch size	Full-batch (entire training set per epoch)
Optimizer	Adam
Learning rate	$10^{-3}$
Epochs	500 epochs per α
Initialization	PyTorch 2.5.1 default Kaiming-uniform (linear layers)
Regularization	None (no weight decay, no explicit $L_{1}/L_{2}$)
α sweep (Pareto tracing)	101 uniformly spaced values in [0,1] (step 0.01)
Numeric precision	FP32 tensors/parameters; channels stored as complex64
Model complexity	$1.70\times 10^{5}$ FLOPs per inference
Inference latency (FP32)	0.8694 ms ± 0.0344 ms (GPU/CUDA)
Hardware	NVIDIA GeForce RTX 2080 Ti (CUDA 12.7); Intel Core i9-9900K CPU
Models trained	One model per α

## Data Availability

The original contributions presented in this study are included in the article. Further inquiries can be directed to the corresponding author.

## Abbreviations

The following abbreviations are used in this manuscript:

AE	Autoencoder
Adam	Adaptive Moment Estimation
BS	Base Station
CNN	Convolutional Neural Network
CR	Communication Rate
CSI	Channel State Information
CU	Communication User
DFRC	Dual-Function Radar-Communication
DL	Deep Learning
DoA	Direction of Arrival
FDSAC	Frequency-Division Sensing-and-Communication
ISAC	Integrated Sensing and Communication
LSTM	Long Short-Term Memory
MF	Matched Filter
MIMO	Multiple-Input Multiple-Output
MISO	Multiple-Input Single-Output
MUSIC	MUltiple SIgnal Classification
RCS	Radar Cross Section
ReLU	Rectified Linear Unit
SE	Squeeze-and-Excitation
SNR	Signal-to-Noise Ratio
SR	Sensing Rate
ULA	Uniform Linear Array
UWB	Ultrawideband
